# Aberrant expressions of *miRNA-206* target, *FN1*, in multifactorial Hirschsprung disease

**DOI:** 10.1186/s13023-018-0973-5

**Published:** 2019-01-07

**Authors:** Gunadi, Nova Yuli Prasetyo Budi, Alvin Santoso Kalim, Wiwid Santiko, Fuad Dheni Musthofa, Kristy Iskandar, Akhmad Makhmudi

**Affiliations:** 1 Pediatric Surgery Division, Department of Surgery, Faculty of Medicine, Public Health and Nursing, Universitas Gadjah Mada/Dr. Sardjito Hospital, Jl. Kesehatan No. 1, Yogyakarta, 55281 Indonesia; 2 Department of Child Health, Faculty of Medicine, Public Health and Nursing, Universitas Gadjah Mada/UGM Academic Hospital, Yogyakarta, 55291 Indonesia

**Keywords:** *FN1*, Hirschsprung disease, Indonesia, *miRNA-206*, *PAX3*, *SDPR*

## Abstract

**Background:**

*MicroRNAs* (*miRNAs*) have been associated with the Hirschsprung disease (HSCR) pathogenesis, however, the findings are still inconclusive. We aimed to investigate the effect of *miRNA-206* and its targets, *fibronectin 1 (FN1), serum deprivation response (SDPR),* and *paired box 3 (PAX3)* expressions on multifactorial HSCR in Indonesia, a genetically distinct group within Asia.

**Methods:**

We determined the *miRNA-206*, *FN1, SDPR* and *PAX3* expressions in both the ganglionic and aganglionic colon of HSCR patients and control colon by quantitative real-time polymerase chain reaction (qRT-PCR).

**Results:**

Twenty-one sporadic HSCR patients and thirteen controls were ascertained in this study. The *miRNA-206* expression was up-regulated (2-fold) in the ganglionic colon and down-regulated (0.5-fold) in the aganglionic colon compared to the control group (ΔC_T_ 12.4 ± 3.0 vs. 14.1 ± 3.9 vs. 13.1 ± 2.7), but these differences did not reach significant levels (*p* = 0.48 and *p* = 0.46, respectively). Interestingly, the *FN1* expression was significantly increased in both the ganglionic (38-fold) and aganglionic colon (18-fold) groups compared to the control group ΔC_T_ 5.7 ± 3.0 vs. 6.8 ± 2.3 vs. 11.0 ± 5.0; *p* = 0.001 and *p* = 0.038, respectively). Furthermore, the expressions of *SDPR* were similar in the ganglionic, aganglionic and control colon groups (ΔC_T_ 2.4 ± 0.6 vs. 2.2 ± 0.4 vs. 2.1 ± 0.6; *p* = 0.16 and *p* = 0.39, respectively), while no change was observed in the *PAX3* expression between the ganglionic, aganglionic, and control colon groups (ΔC_T_ 3.8 ± 0.8 vs. 4.1 ± 0.8 vs. 3.7 ± 1.1; *p* = 0.83 and *p* = 0.44, respectively).

**Conclusion:**

Our study is the first report of aberrant *FN1* expressions in the colon of patients with HSCR and supplies further insights into the contribution of aberrant *FN1* expression in the HSCR pathogenesis.

## Background

Hirschsprung disease (HSCR: MIM# 142623) is a complex genetic disorder characterized by the absence of ganglion cells in the intestines, resulting in a functional obstruction in children. HSCR is classified as follows: short-segment HSCR, long-segment HSCR, and total colonic aganglionosis [1, 2]. The incidence of HSCR varies among ethnic groups with 1.5, 2.1, and 2.8 cases per 10,000 live births in European, African and Asian ancestry cases, respectively [1, 2].

At least 15 genes have been associated with the pathogenesis of HSCR, with the *RET* gene as primarily responsible for HSCR [1, 2]. However, the majority of those genes make minor contributions to HSCR [3–5]. Recent studies have proposed some *microRNAs* (*miRNAs*) targets contribute important roles in the pathogenesis of HSCR, but the findings are still inconclusive [6–8]. *miRNA* is a small non-coding RNA that deregulates gene expression at the posttranscriptional level. It is stable and easily measureable in the patients’ tissue and blood specimens, including HSCR patients’ colon [6–8].

*miRNA-206* has been shown to be down-regulated and targeted three genes, named *fibronectin 1 (FN1), serum deprivation response (SDPR)*, and *paired box 3 (PAX3)*, in HSCR patients in Chinese population [7]. In addition, some genetic differences might exist among Asian population [9] and our previous study revealed that the impact of *SEMA3* rs11766001 variant differs among ethnic groups[10]. Therefore, we aimed to investigate the expressions of *miRNA-206* and its targets, *FN1, SDPR*, and *PAX3*, in HSCR patients in Indonesia, a genetically distinct group within Asia.

## Material and methods

### Patients

This study was conducted at Dr. Sardjito Hospital, a referral and academic hospital in Yogyakarta, Indonesia. All children with the age of < 18 years old with diagnosis of HSCR according to clinical findings, contrast enema and histopathology were involved in this study, except those that had low quality of total RNA [4, 5, 10–12].

The ganglionic and aganglionic colon of HSCR patients were collected at definitive surgery, while the control colon samples were obtained at stoma closure from anorectal malformation patients [12].

A written informed consent was signed by the HSCR patients’ and control parents to ascertain this study. The Institutional Review Board of the Faculty of Medicine, Public Health and Nursing, Universitas Gadjah Mada/Dr. Sardjito Hospital gave approval for this study (KE/FK/786/EC/2015).

### Total RNA isolation and quantitative real-time polymerase chain reaction (qRT-PCR)

The miRCURY™ RNA Isolation Kit-Tissue (Exiqon A/S, Denmark) was used to extract the total RNA from colon tissue. Subsequently, the total RNA was measured using a NanoDrop 2000 Spectrophotometer (Thermo Scientific, Wilmington, DE, USA). Only high quality RNAs with the OD260/280 ratios of 1.8 to 2.0 were utilized for the subsequent experiment.

The qRT-PCR was performed to determine the expression of *miRNA-206, FN1, SDPR*, and *PAX3* using the BioRad CFX Real-Time PCR System (California, USA), the Universal cDNA Synthesis Kit II (Exiqon A/S, Denmark), ExiLENT SYBR® Green Master Mix Kit (Exiqon A/S, Denmark), and miRCURY™ LNA™ Universal RT microRNA PCR System (Exiqon A/S, Denmark). *U6 small nuclear RNA (snRNA)* served as a control for analysis of *miRNA-206* expression, while *glyceraldehyde-3-phosphate dehydrogenase (GAPDH)* was utilized as a reference gene for analysis of *FN1*, *SDPR, and PAX3* expression. All qRT-PCR reactions were performed in duplicate.

The *hsa-miRNA-206 and U6 snRNA* primers were 5’-ACGAGTTTAGAGCCGGATAGCCACACAC-3′ (RT), 5’-TGACGAGTTTAGAGCCGGATAG-3′ (forward), and 5’-GCGTTGTCTGGAATGTAAGGAAGT -3′ (reverse); and 5’-CTCGCTTCGGCAGCACA-3′ (forward) and 5’-AACGCTTCACGAATTTGCGT-3′ (reverse), respectively [13], while the primer sequence for *FN1*, *SDPR*, *PAX3*, and *GAPDH* were 5′-CAAGCCAGATGTCAGAAGC-3′ (forward) and 5′-GGATGGTGCATCAATGGCA-3′ (reverse); 5′-AGTCACGGTGCTCACGCTCC-3′ (forward) and 5′- GTTGCTGGTGGAGGCCTGGT-3′ (reverse); 5'-ACCACCTTCACAGCAGAACA-3' (forward) and 5'-CAGCTTGCTTCCTCCATCTT-3' (reverse); and 5′-GCACCGTCAAGGCTGAGAAC-3′ (forward) and 5′-TGGTGAAGACGCCAGTGGA-3′ (reverse), respectively [12, 14–17].

The Livak (2^-ΔΔC^_T_) method was used to analyze the *miRNA-206, FN1*, *SDPR,* and *PAX3* expression level [18].

### Statistical analysis

The *miRNA-206, FN1*, *SDPR,* and *PAX3* expressions were determined as mean values ± standard deviation (SD) and t-tests were used to determine any statistical differences between the ganglionic and aganglionic colon of HSCR patients and control groups. A *p*-value < 0.05 was considered statistically significant.

## Results

We obtained twenty-one colon samples from sporadic non-syndromic HSCR patients, of whom 12 and 9 were males and females, respectively, and thirteen colon specimens from non-HSCR patients. Most (90%) patients had short-segment HSCR and underwent transanal endorectal pull-through (76%) (Table 1).

**Table 1:** Clinical characteristics of Indonesian HSCR patients involved in this study

Characteristic	n (%); months ± SD
Gender	
Male	12 (57)
Female	9 (43)
Aganglionosis type	
Short	19 (90)
Long	2 (10)
Total colon aganglionosis	0
Age at diagnosis	14.3 ± 31.2
Colostomy	5 (28)
Age at definitive surgery	22.1 ± 34.1
Definitive surgery	
Transanal endorectal pull-through	16 (76)
Duhamel	3 (14)
Soave	2 (10)

**Fig. 1:**
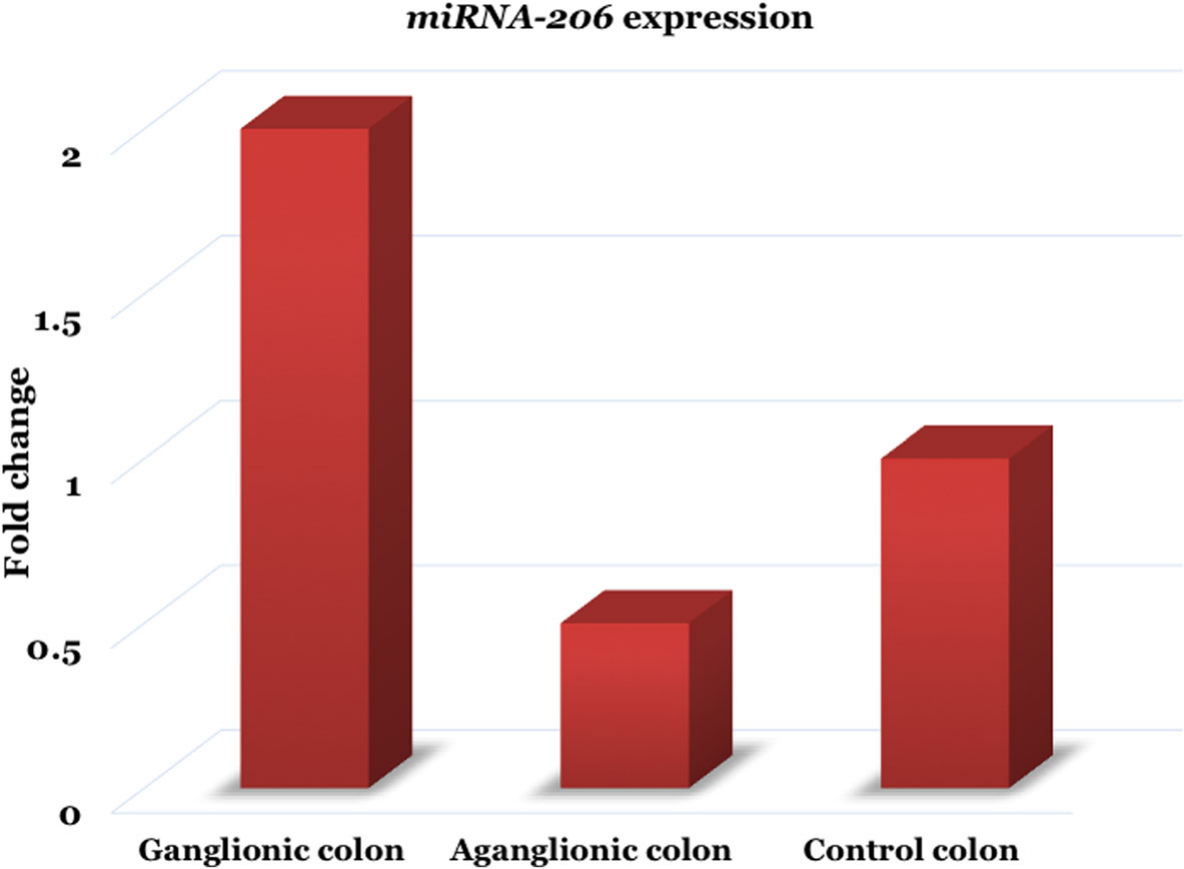
The *miRNA-206* expression was up-regulated (2-fold) in the ganglionic colon and down-regulated (0.5-fold) in the aganglionic colon compared to the control group, but these differences did not reach significant level

Although the *miRNA-206* expression was up-regulated (2-fold) in the ganglionic colon and down-regulated (0.5-fold) (Fig. 1) in the aganglionic colon compared to the control group (ΔC_T_ 12.4 ± 3.0 vs. 14.1 ± 3.9 vs. 13.1 ± 2.7), but these differences did not reach significant levels (*p* = 0.48 and *p* = 0.46, respectively) (Table 2).

**Table 2:** The *miRNA-206* expression in both the ganglionic and aganglionic colon of HSCR patients and control colon

	ΔC_T_ ± SD	ΔΔC_T_ (95% CI)	2^-ΔΔC^_T_ (Fold change)	*p*-value
Ganglionic colon	12.4 ± 3.0	−0.8 (−3.0–1.4)	2.0	0.48
Aganglionic colon	14.1 ± 3.9	1.0 (− 1.7–3.6)	0.5	0.46
Control colon	13.1 ± 2.7			

**Fig. 2:**
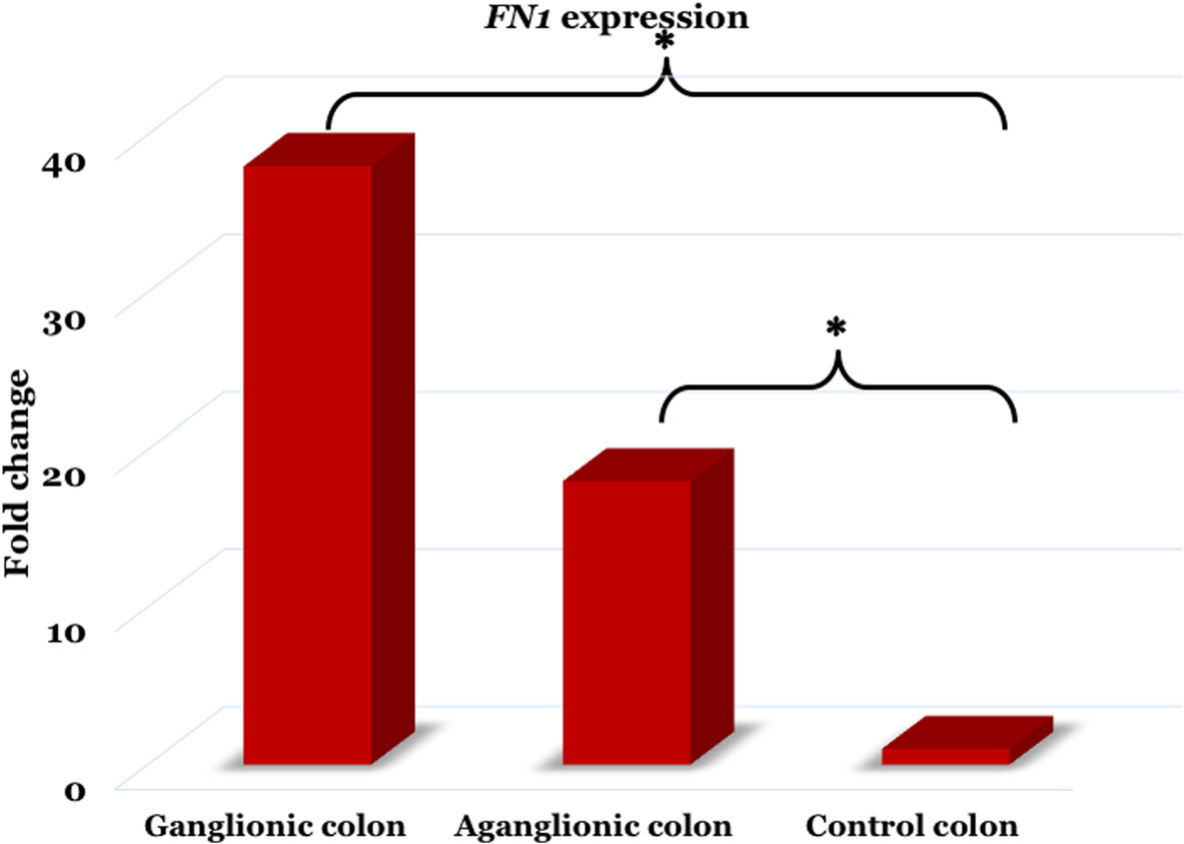
The *FN1* expression was increased in both the ganglionic (38-fold) and aganglionic colon (18-fold) groups compared to the control group, with *p*-value of 0.001 and 0.038, respectively. *, *p* < 0.05

Interestingly, the *FN1* expression was significantly up-regulated in both the ganglionic (38-fold) and aganglionic colon (18-fold) (Fig. 2) groups compared to the control group (ΔC_T _5.7 ± 3.0 vs. 6.8 ± 2.3 vs. 11.0 ± 5.0; *p* = 0.001 and *p* = 0.038, respectively) (Table 3).

**Table 3:** The *FN1* expression in both the ganglionic and aganglionic colon of HSCR patients and control colon

	ΔC_T_ ± SD	ΔΔC_T_ (95% CI)	2^-ΔΔC^_T_ (Fold change)	*p*-value
Ganglionic colon	5.7 ± 3.0	−5.3 [− 8.2 – (−)2.3]	38	0.001*
Aganglionic colon	6.8 ± 2.3	−4.1 [− 8.1 – (−)0.2]	18	0.038*
Control colon	11.0 ± 5.0			

*, *p* < 0.05 is considered statistically significant

Furthermore, the expressions of *SDPR* were similar in the ganglionic, aganglionic and control colon groups (ΔC_T_ 2.4 ± 0.6 vs. 2.2 ± 0.4 vs. 2.1 ± 0.6; *p* = 0.16 and *p* = 0.39, respectively) (Table 4), while no change was observed in the *PAX3* expression between the ganglionic, aganglionic, and control colon groups (ΔC_T_ 3.8 ± 0.8 vs. 4.1 ± 0.8 vs. 3.7 ± 1.1; *p* = 0.83 and *p* = 0.44, respectively) (Table 5).

**Table 4:** The *SDPR* expression in both the ganglionic and aganglionic colon of HSCR patients and control colon

	ΔC_T_ ± SD	ΔΔC_T_ (95% CI)	2^-ΔΔC^_T_ (Fold change)	*p*-value
Ganglionic colon	2.4 ± 0.6	0.3 (−0.1–0.8]	0.8	0.16
Aganglionic colon	2.2 ± 0.4	0.2 (− 0.2–0.6)	0.9	0.39
Control colon	2.1 ± 0.6

**Table 5:** The *PAX3* expression in both the ganglionic and aganglionic colon of HSCR patients and control colon

	ΔC_T_ ± SD	ΔΔC_T_ (95% CI)	2^-ΔΔC^_T_ (Fold change)	*p*-value
Ganglionic colon	3.8 ± 0.8	0.1 (−0.9–1.1)	0.9	0.83
Aganglionic colon	4.1 ± 0.8	0.4 (− 0.7–1.5)	0.8	0.44
Control colon	3.7 ± 1.1			

## Discussion

We describe new data on the *miRNA-206* expression in Indonesian HSCR patients. We were unable to find evidence of the impact of *miRNA-206* in the pathogenesis of HSCR in Indonesian population, although its expression was ~ 2-fold up-regulated and ~ 0.5-fold down-regulated (Fig. 1) in the ganglionic and the aganglionic colon of HSCR patients, respectively, compared to the control colon. These results are different with previous report [7]. It has been shown that the *miRNA* expression significantly differed between two populations, CEU (Utah residents with northern and western European ancestry) and YRI (Yoruba people from Ibadan, Nigeria) [19]. In addition, *miRNA-26a* expression was also different between the prostate cancer cell lines derived from African American ancestry and those derived from Caucasian ancestry [20]. Interestingly, the population differences in *miRNA* expression are affected by genetic variants [19]. Therefore, the *miRNA-206* expression differences between previous report and our study might relate to Indonesian genetic structure ethnicity [9, 10].

The down-regulation of *miRNA-206* has been hypothesized to be involved in the pathogenesis of HSCR patient through the *SDPR* up-regulation resulting in the deformation of the caveolae of neural crest cells in the intestines [7]. Our study reveals a new evidence opposing this hypothesis by providing data from a population genetically different from previous study [7]. However, our results should be interpreted with some caution since our study had a different approach from the previous report [7]; we determined the *miRNA-206* expression in the colon tissue using RT-PCR only (vs. they also performed in vitro study employing the human 293 T and SH-SY5Y cell lines). Also, it should be noted the main weakness of our study is the small sample size, which suggests that a larger sample size needs to be involved to clarify and confirm our results.

Although several *miRNAs* have been shown to have a role in HSCR pathogenesis, however, the evidence for actual etiology remains inconclusive [6–8]. Therefore, in the meanwhile, it is always challenging to determine which *miRNAs* have the strongest impact on the HSCR pathogenesis. Those *miRNAs* may serve as potential biomarkers and/or molecular therapy for patients with HSCR in the future since the *miRNAs* are stable and easily measureable in the patients’ tissue and blood specimens.

Moreover, our study showed that the expression of *PAX3* did not differ between the HSCR and the control groups. *PAX3* has been associated with syndromic HSCR, i.e. Waardenburg syndrome [21]. Our cohort patients are non-syndromic HSCR, therefore, it might be important to conduct a study involving the syndromic HSCR to clarify the results.

Intriguingly, the expression of *FN1* was strongly up-regulated in both the ganglionic and aganglionic colon of HSCR patients compared to the control colon. To the best of our knowledge, this report is the first study of aberrant *FN1* expressions in the colon of HSCR patients. It has been shown that *FN1* is up-regulated by enteric glial cells in the proliferating intestinal epithelial cells [22]. HSCR is a developmental defect of the enteric nervous system (ENS). HSCR pathogenesis might involve the compromised condition of genes responsible for gangliogenesis of the ENS [1–4] and/or their interactions [1, 2, 5, 23]. Furthermore, integration of different pathways synchronizing neurogenesis and gliogenesis is also important for the proper development of ENS and defects in any of these signaling elements might result in HSCR [24, 25]. Gui et al. showed that *GDNF* stimulates neuronal differentiation, while *NRG1* strongly induces the glial differentiation of enteric neural crest cells (ENCCs) [24], whereas Ngan et al. revealed that *Ptch1* knockout in mouse ENCCs promotes up-regulated *Dll1* expression and stimulates the *Notch* signaling, resulting in a premature gliogenesis and reduced ENCC progenitors in intestines [25]. Therefore, further in vitro assay of *FN1* knockdown in primary culture of ganglion (mixture of neurons and glial cells) are necessary to see the effect of *FN1* knockdown on the proliferation, differentiation and survival of both neurons and glial cells, and the balance of neurogenesis and gliogenesis. Unfortunately, we do not have any data on in vitro assay of *FN1* knockdown in primary culture of ganglion due to resource limitations in our laboratory.

## Conclusion

Our study is the first report of aberrant *FN1* expressions in the colon of patients with HSCR and supplies further insights into the contribution of aberrant *FN1* expression in the HSCR pathogenesis.

## Abbreviations

FN1: Fibronectin 1
GAPDH: Glyceraldehyde-3-phosphate dehydrogenase
HSCR: Hirschsprung disease
miRNA: MicroRNA
PAX3: Paired box 3
qRT-PCR: Quantitative real-time polymerase chain reaction
SDPR: Serum deprivation response
snRNA: Small nuclear RNA
